# Herpes Zoster Oticus With Facial Palsy and Hearing Loss: A Case Report of Ramsay Hunt Syndrome in an Older Patient

**DOI:** 10.1002/ccr3.70272

**Published:** 2025-02-24

**Authors:** James Kakeeto, Ronaldine Anne Atukunda, Priscilah Asio, Ivaan Pitua

**Affiliations:** ^1^ School of Medicine, College of Health Sciences Makerere University Kampala Uganda

**Keywords:** facial nerve palsy, hearing loss, herpes zoster Oticus, Ramsay Hunt syndrome

## Abstract

Ramsay Hunt syndrome (RHS) is a rare complication of varicella‐zoster virus (VZV) reactivation, typically presenting with a combination of painful vesicular lesions in the ear, facial nerve palsy, and hearing loss. Early diagnosis and antiviral therapy are critical in minimizing long‐term sequelae, such as persistent facial weakness and hearing impairment. This case highlights the importance of prompt recognition and treatment in older patients, particularly those with pre‐existing hearing difficulties. An 87‐year‐old female presented with a 2‐week history of painful, blister‐like lesions on the right ear and lips, associated with a burning sensation. Over several days, the lesions progressed to visible wounds. The patient also reported increasing weakness, difficulty eating, and worsening hearing impairment. Physical examination revealed vesicular lesions on the right pinna and lips, along with right‐sided facial weakness (House‐Brackmann Grade IV) and slight ipsilateral lagophthalmos with mild conjunctival erythema. No new vesicles or generalized rash were observed. The patient's medical history included hypertension and long‐standing bilateral hearing difficulty. Ramsay Hunt syndrome was diagnosed based on the characteristic symptoms of painful vesicular lesions, facial nerve palsy, and hearing loss. The patient was treated with oral acyclovir, dexamethasone, and supportive care. Significant improvement was noted by Day 7, with reduced pain, partial recovery of facial function, and improvement in hearing. By Day 14, all lesions had healed, and facial symmetry was restored. This case emphasizes the importance of early recognition and intervention in improving clinical outcomes in older patients with RHS.

**Summary**

- Early recognition of Ramsay Hunt syndrome in older patients with painful vesicular lesions, facial nerve palsy, and worsening hearing loss is crucial for effective management.
- Timely antiviral therapy and corticosteroids can significantly improve outcomes, preventing long‐term complications such as persistent facial weakness and permanent hearing impairment.

## 1 Introduction

Ramsay Hunt syndrome (RHS) was first identified in 1907 by neurologist James Ramsay Hunt. This rare condition is characterized by Herpes Zoster Oticus (HZO) and peripheral facial paralysis on the same side, resulting from the reactivation of the Varicella Zoster Virus (VZV) within the geniculate ganglion [1, 2]. The initial VZV infection causes fever and a widespread vesicular rash, commonly known as chickenpox. After the primary infection, the virus can remain dormant in the body. When reactivated, it leads to “zoster” or “herpes zoster,” which is marked by pain and a vesicular rash along the affected nerve's distribution, typically confined to a single dermatome. The specific symptoms and rash distribution depend on the nerve involved. In less than 1% of zoster cases, the facial nerve is affected, resulting in RHS [3, 4]. HZO is characterized by vesicular eruptions on one side, affecting the pinna, external ear canal, and tympanic membrane, often following intense ear pain (otalgia). The virus may also affect the vestibular, trigeminal, and cochlear nerves, leading to various cranial nerve abnormalities [5, 6]. Cranial polyneuritis, a rare complication of RHS, occurs in 1.8% of patients and is frequently accompanied by symptoms such as hearing loss and balance disturbances. The associated symptoms can vary between individuals, depending on their overall health and the route of infection, and they may appear before or after the primary symptoms of RHS [7]. This variability complicates the diagnosis, often leading to delays in treatment. Therefore, early diagnosis and timely medical intervention are critical for patients with RHS, considering the spontaneous recovery rate and the potential for long‐term complications.

## 2 Case History/Examination

An 87‐year‐old female presented with a 2‐week history of painful, blister‐like lesions on the right ear (pinna) and lips, which were preceded by a burning sensation. Over several days, the lesions progressed to visible wounds. The patient initially sought treatment at a peripheral clinic, where she was prescribed unknown medications but experienced no improvement. Four days prior to admission, she reported increasing weakness, difficulty eating, and worsening hearing difficulty. Her medical history included hypertension, managed with amlodipine 10 mg daily, and long‐standing hearing difficulty that seemed to worsen with the onset of her present symptoms. One year earlier, she had experienced right‐sided facial deviation and pain, which resolved with NSAID treatment and no further intervention.

**FIGURE 1:**
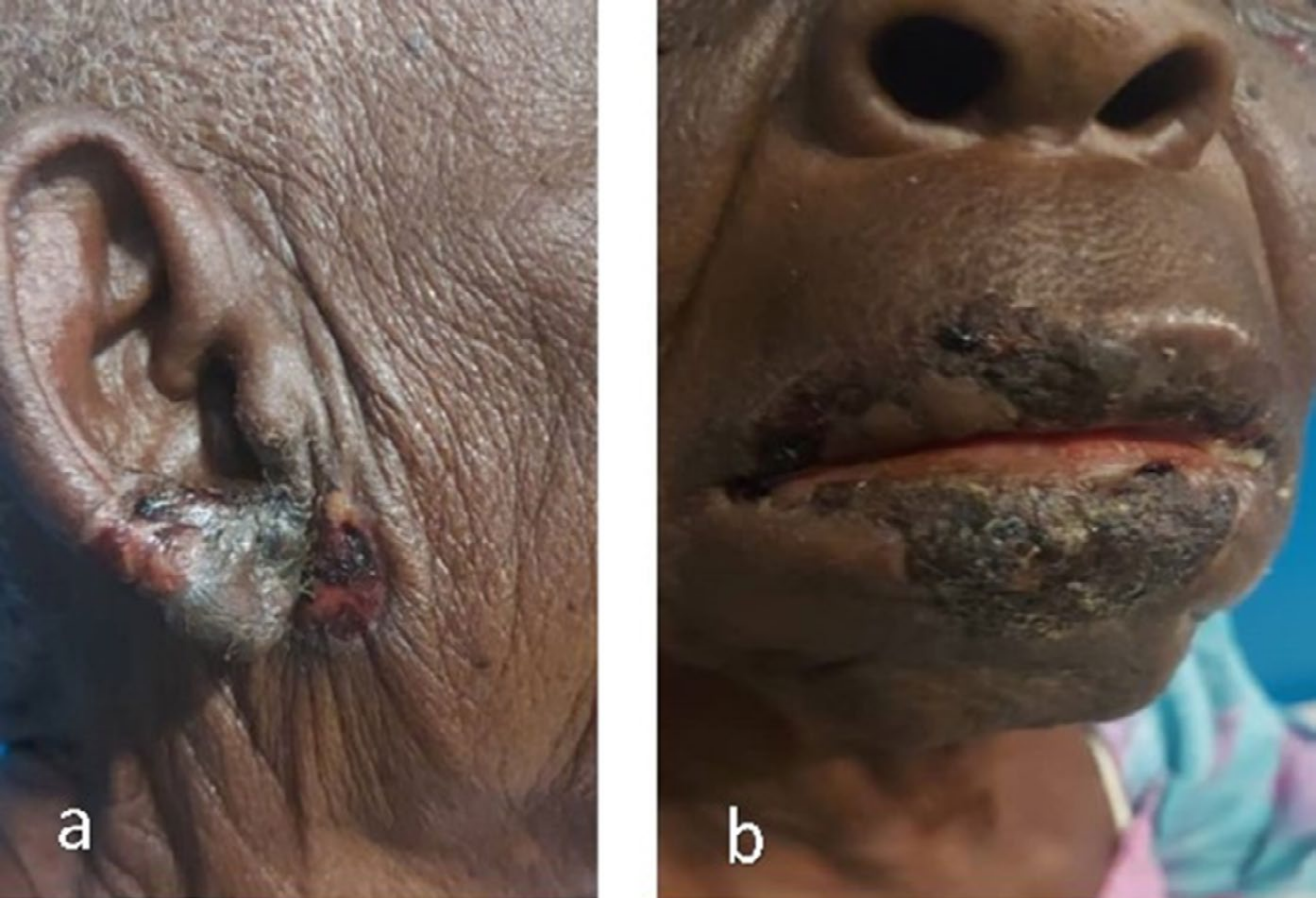
Multiple crusted vesicular lesions on the pinna (a) and lips (b) with dark scabbing.

On examination, multiple crusted vesicular lesions were observed on the right pinna and lips. The lip lesions showed erosions and dark scabbing (Figure 1). The right pinna and auditory canal exhibited erythematous, crusted lesions, consistent with HZO (Figure 1). The patient also demonstrated right‐sided facial weakness with deviation of the mouth to the left, indicating House‐Brackmann Grade IV facial nerve dysfunction. Additionally, she had lagophthalmos and mild conjunctival erythema of the right eye. No new vesicles or generalized rash were noted. The patient complained of a perception of worsening hearing loss, which was affecting her daily interactions, her auditory function was assessed using a tuning fork, as advanced audiological equipment such as pure tone audiometry was unavailable at and around the remote medical center in Uganda. Weber's test revealed lateralization to the unaffected left ear, a finding consistent with sensorineural hearing loss on the right side. Rinne's test on the right ear showed air conduction louder than bone conduction (a positive result), further supporting SNHL as there was no evidence of conductive hearing loss. These results, combined with the clinical features of RHS, including vesicular lesions, facial nerve palsy, and otalgia, strongly supported the diagnosis of SNHL on the affected right side.

## 3 Differential Diagnosis

The differential diagnosis for this case included; Bell's palsy, given the facial weakness and asymmetry, Bell's palsy was considered; however, the presence of vesicular lesions on the right ear and lip, as well as the associated hearing loss, made this diagnosis less likely. Herpes simplex virus (HSV) Infection could present with similar facial lesions, but the distinctive involvement of the ear and hearing loss pointed toward herpes zoster. Acute Otitis Media or Externa with Facial Nerve Palsy, although facial nerve involvement can occur in otitis, the vesicular rash localized to the ear and lip was not consistent with this diagnosis. So, the combination of painful vesicular lesions in the external auditory canal and pinna, ipsilateral facial nerve palsy, and hearing loss led to the diagnosis of RHS.

## 4 Discussion

The current case involved a classic presentation of RHS in an older female patient, laying emphasis on the role of early recognition and timely treatment. Our diagnosis of RHS was strongly supported by the presence of characteristic symptoms, including the vesicular rash localized to the external ear and lips and ipsilateral facial nerve palsy. Additionally, the patient exhibited hearing loss, a hallmark feature of RHS, which can be caused by the involvement of the cochlear nerve or, in some cases, the vestibular nerve [6]. These findings, alongside the patient's medical history, pointed toward RHS as the most likely diagnosis while ruling out other potential causes of facial nerve palsy.

Timely diagnosis and intervention are critical in managing RHS to prevent long‐term complications such as persistent facial weakness and complete hearing loss. In this case, the patient was promptly treated with antiviral therapy (oral acyclovir) and corticosteroids (dexamethasone), which are the cornerstone treatments for RHS. Antiviral therapy is most effective when initiated within 72 h of symptom onset and is known to reduce the severity and duration of the disease, while corticosteroids help decrease inflammation and improve recovery of facial nerve function [8]. In this patient, treatment began after a 2‐week history of symptoms, which is somewhat delayed, but she showed marked improvement after 7 days of therapy, with significant recovery in facial nerve function and partial improvement in hearing. By Day 14, all vesicular lesions had healed, and the patient regained facial symmetry, highlighting the importance of prompt intervention even if the diagnosis is initially delayed.

The clinical presentation and treatment approach in this case are consistent with findings from existing literature on RHS. Early antiviral therapy is crucial for improving outcomes, particularly in older patients, as delayed treatment is associated with a higher risk of long‐term sequelae such as persistent facial weakness and hearing loss [1, 2, 6, 7]. Other studies have highlighted the importance of distinguishing RHS from other conditions that can present with similar symptoms, including Bell's palsy, HSV infection, and otitis externa or media [9, 10]. A study emphasized that older patients, particularly those with pre‐existing hearing loss, may present with atypical features of RHS, making early recognition even more challenging but crucial for optimal treatment [5, 11]. Our case points out the necessity of considering RHS in older patients presenting with unilateral facial paralysis, painful ear lesions, and hearing loss especially in resource limited settings. Based on Weber's and Rinne's test results and the patient's subjective complaints of worsening hearing affecting her daily interactions, the hearing loss on the right side was consistent with at least moderate sensorineural hearing impairment. Advanced audiological evaluations to quantify the exact degree of hearing loss were unavailable due to resource limitations. However, prompt diagnosis and appropriate antiviral and corticosteroid therapy can significantly improve clinical outcomes and prevent the development of permanent disabilities, such as persistent facial nerve palsy and complete hearing impairment.

## 5 Conclusion and Results

**FIGURE 2:**
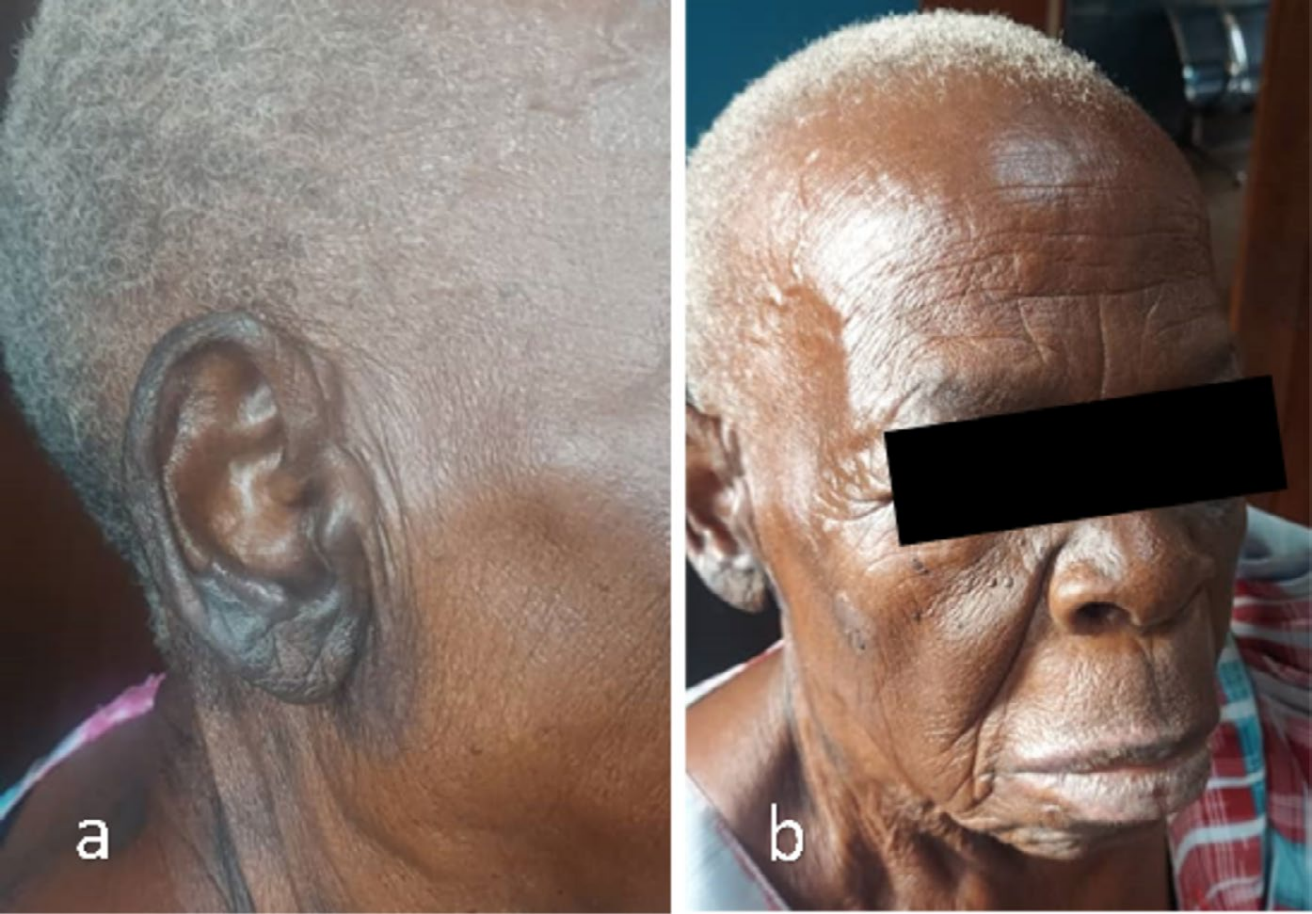
Day 14, completely healed pinna with residual hyperpigmentation (a), no signs of wounds or lesions on the lips (b).

The patient was diagnosed with RHS based on the clinical presentation, which included painful vesicular lesions on the right pinna and lip, right‐sided facial nerve palsy, and worsening hearing loss. The patient was admitted for supportive care and antiviral treatment. She was given Normal saline (2 L/day with 10% dextrose in ratio of 1:1), intravenous paracetamol 1 g every 8 h for pain, dexamethasone 8 mg once daily for 3 days, and oral acyclovir 800 mg five times a day for 7 days. At discharge on Day 3, the patient showed slight improvement, with reduced pain and the vesicular lesions beginning to crust. By the first review on Day 7, significant progress was evident: the lesions had dried and scabbed, and facial weakness had improved to House‐Brackmann Grade II, with partial recovery of her smile, ability to eat, and to close her right eye. Hearing showed partial improvement noted subjectively. By Day 14, all lesions had healed, and the patient's facial symmetry was restored, with no signs of asymmetry (Figure 2). There were no complaints of residual pain, and no new lesions had developed. The timely initiation of antiviral therapy and supportive care was key to the patient's recovery.

## Author Contributions

**James Kakeeto:** conceptualization, data curation, formal analysis, investigation, methodology, project administration, resources, software, writing – original draft, writing – review and editing. **Ronaldine Anne Atukunda:** methodology, writing – original draft, writing – review and editing. **Priscilah Asio:** methodology, writing – original draft, writing – review and editing. **Ivaan Pitua:** conceptualization, data curation, investigation, methodology, supervision, validation, writing – original draft, writing – review and editing.

## Ethics Statement

Written informed consent was acquired from the patient for participation in the case report and publication in a peer‐reviewed journal.

## Consent

Written informed consent for publication of this case report and accompanying images was obtained from the patient.

## Conflicts of Interest

The authors declare no conflicts of interest.

## Data Availability

All relevant data and materials are available within the text.
